# New Drugs, Appliances, and Things Medical

**Published:** 1890-03-29

**Authors:** 


### NEW DRUGS, APPLIANCES, AND THINGS MEDICAL.

[All preparations, appliances, novelties, etc., of which a notice is desired, should be sent to The Editor, The Lodge, Porchester Square, W.]

### ROBINSON'S PATENT GROATS AND PATENT BARLEY.

These well-known foods have been before the public for seventy years, and consequently want no praise on our part to establish the purity of manufacture or value as food stuffs. Dr. W. Johnstone's analyses are as follows :—

| | Pat. Groats. Per cent. | Pat. Barley |
|---|---|---|
| Hydroscopic moisture | 6·77 | 9·18 |
| Fatty matters | 8·18 | 0·95 |
| Albumenous compounds or flesh formers | 7·00 | 4·37 |
| Carbohydrates or heat producers | 75·70 | 84·01 |
| Cellulose | 0·95 | 0·74 |
| Mineral matter (ash) | 1·40 | 0·75 |
| Total | 100·00 | 100·00 |

These analyses show that the above are first-class specimens of manufactured oat and barley meals. We have carefully examined them both microscropically and qualitatively. The result of our investigation is to prove the absence of all deleterious material and the perfect purity of the products. The patent groats make most excellent porridge and gruel, and the patent barley formed the foundation of the best barley-water we have ever tasted. We consider that the aqueous solution of the barley meal forms a most desirable diluent for milk where extra carbohydrate food is required for infants. Our cook speaks highly of the groats for oat cakes and the barley for puddings and barley cake.

### THE SANITARY WOOD-WOOL COMPANY'S PREPARATIONS.

The secretary of the above company has forwarded to us samples of the special preparations made by the company. We have used these for some time, and found them of great service. We are particularly pleased with the accouchement sheets and diapers or sanitary towels. They are most absorbent and cleanly, and possess the useful property of cheapness, so that after use they can be put on the fire, and so all chance of retaining infection is done away with. The wood-wool wadding and tissue are both good forms of wound dressings, and as they are "sublimated" their antiseptic qualities are guaranteed. The tissue we have found most useful, not only for wound-dressing and absorbent purposes generally, but for splint-packing, and making the first layer of plaster of Paris fixation apparatus. Nurses have informed us that the sanitary towels for ordinary use are everything that can be desired.

